# The dual role of activating transcription factor 4: from cellular stress sentinel to cardiovascular disease intervention

**DOI:** 10.7717/peerj.20494

**Published:** 2026-01-16

**Authors:** Yaping Wang, Jie Yuan, Feifan Wang, Hong Ma

**Affiliations:** 1 Department of Endocrinology, The Second Affiliated Hospital, Zhejiang University School of Medicine, Hangzhou, Zhejiang, China; 2 Department of Urology, Sir Run Run Shaw Hospital, Zhejiang University School of Medicine, Hangzhou, Zhejiang, China; 3 Department of Cardiology, The Second Affiliated Hospital, Zhejiang University School of Medicine, State Key Laboratory of Transvascular Implantation Devices, Hangzhou, Zhejiang, China

**Keywords:** ATF4, Integrated stress response, Oxidative stress, Ferroptosis, Serine biosynthesis, Endoplasmic reticulum stress

## Abstract

As the master orchestrator of integrated stress response, activating transcription factor 4 (ATF4) operates as a central molecular switch that directs cellular fate toward survival or death by regulating genes associated with oxidative stress, endoplasmic reticulum stress, apoptosis, ferroptosis and metabolism. The functional outcome of ATF4 activation is critically dependent on the context: it usually contributes to cellular adaption and survival under mild or transient stress, yet triggers cell death when stress is severe or prolonged. Dysregulation of this dichotomous function has been implicated in a variety of diseases, such as cancer, neurodegenerative disease, metabolic disease, *etc.,* highlighting ATF4 as a potential therapeutic target. Recently, growing evidence has further underscored the dual roles of ATF4 as the guardian or executioner in cardiovascular disorders, such as coronary heart disease, cardiomyopathy, arrhythmia, valvular heart disease, heart failure and cardiovascular aging. Here in this review, we systematically decode the context-dependent opposing roles of ATF4 in cardiovascular diseases and also highlight the underlying regulatory mechanisms, thereby providing a rationale for developing context-specific therapeutic strategies targeting ATF4 for the personalized management of cardiovascular disorders.

## Introduction

Cardiovascular disease ranks first among the causes of death, accounting for approximately one third of deaths worldwide (GBD 2017 Causes of Death Collaborators, 2018). It is estimated that this trend will continue to rise with the epidemic of obesity, metabolic syndrome, physical inactivity and aging (Soppert et al., 2020). Thus, to further elucidate the pathological mechanisms and explore effective prevention and therapeutic strategies are of great significance for relieving the global burden of cardiovascular diseases.

**Figure 1:**
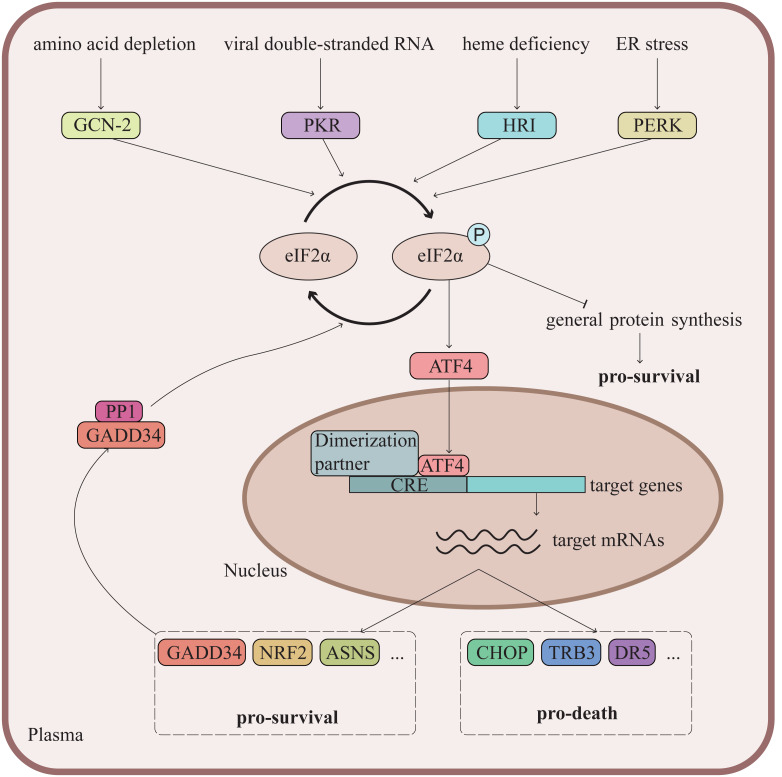
ATF4 is the main effector of cellular integrated stress response (ISR). The ISR is an evolutionarily conserved defense mechanism triggered by various stress conditions, including amino acid deprivation, viral infection, heme deficiency, endoplasmic reticulum (ER) stress, *etc*. These stresses activate general control nonderepressible 2 (GCN-2), double-stranded RNA-dependent protein kinase (PKR), heme-regulated inhibitor (HRI) and PKR-like endoplasmic reticulum kinase (PERK) respectively, which further phosphorylate the α subunit of eukaryotic translation initiation factor 2 (eIF2α). The phosphorylation of eIF2α generally inhibits protein synthesis, but preferentially upregulates the translation of certain mRNAs containing upstream open reading frames, including ATF4, which acts as the major effector of ISR through regulating the transcription of numerous genes. Specifically, ATF4 drives expression of *PPP1R15A* (GADD34), which forms a complex with protein phosphatase 1 (PP1) to dephosphorylate eIF2α. This negative feedback loop terminates ISR and restores protein synthesis. The ISR activation usually contributes to cell adaption and survival in face of challenges, but may also lead to cell death during persistent stresses. CRE, cyclic adenosine monophosphate- response element.

As a cellular adaptive mechanism to cope with various stress challenges, the integrated stress response (ISR) has been reported to play a critical role in the pathogenesis of cardiovascular diseases, such as pathological cardiac hypertrophy, diabetic cardiomyopathy, ischemic cardiomyopathy, *etc*. (Lu, Koju & Sheng, 2024; Ranea-Robles et al., 2022; Wu et al., 2024a). Specifically, there are four kinases in mammalian cells, namely general control nonderepressible 2 (GCN2), double-stranded RNA-dependent protein kinase (PKR), heme-regulated inhibitor (HRI) and PKR-like endoplasmic reticulum kinase (PERK), which sense amino acid depletion, viral infection, heme deficiency and endoplasmic reticulum (ER) stress respectively (Ebert et al., 2022; Yan et al., 2024). Activation of the four kinases all converge to phosphorylation of the α subunit of eukaryotic translation initiation factor 2 (eIF2α), which generally reduces protein synthesis but selectively enhances the translation of specific mRNAs, especially for activating transcription factor 4 (ATF4), which is the most widely studied effector of ISR (Fig. 1) (Yan et al., 2024). This complex cellular network helps the stressed cells to self-repair and restore homeostasis, but may also induce cell death depending on the duration and intensity of the stresses (Tian et al., 2021). The ISR thus governs a dualism in cell fate under different stress conditions. This fate is primarily governed by the dynamics of eIF2α phosphorylation and the downstream signaling of ATF4 (Pakos-Zebrucka et al., 2016). A key regulatory mechanism is the ATF4-driven expression of *PPP1R15A* (GADD34), which forms a complex with protein phosphatase 1 (PP1) to dephosphorylate eIF2α (Pakos-Zebrucka et al., 2016; Ryoo, 2024). This negative feedback loop terminates the ISR, restores protein synthesis, and facilitates recovery once the stress is resolved (Pakos-Zebrucka et al., 2016; Ryoo, 2024). However, when the stress is irreparable, the GADD34-PP1 complex-mediated restoration of protein synthesis paradoxically facilitates the production of death-inducing proteins, thereby executing cell death when homeostasis is unattainable (Pakos-Zebrucka et al., 2016; Ryoo, 2024).

ATF4 is a DNA-binding protein consisting of 351 amino acids and widely expressed in various cells (Ameri & Harris, 2008). The level of ATF4 is relatively low under physiological conditions, but significantly induced in response to cellular stresses (Tang et al., 2024). As a basic leucine zipper (bZIP) transcription factor belonging to the cyclic adenosine monophosphate (cAMP)-response element binding protein family, ATF4 binds to the cAMP-response element sequences of target genes though the basic region, which is located at the N-terminus of the bZIP domain, thereby functioning as an activator or repressor of gene transcription (Ebert et al., 2022; Li et al., 2023). Besides, the leucine zipper region, which is located at the C-terminus of the bZIP domain, is responsible for protein-protein interaction, facilitating ATF4 to assemble into homodimers or heterodimers to exert transcriptional regulatory potentials (Adams, Ebert & Dyle, 2017). Notably, ATF4 is subject to extensive post-translational modifications (PTMs), including ubiquitination, SUMOylation, phosphorylation, and acetylation, which collectively modulate its stability, transcriptional activity, and dimerization selectivity (Neill & Masson, 2023). While ATF4 is a well-established effector downstream of eIF2α phosphorylation, its regulation and functional scope extend far beyond this canonical pathway. By orchestrating the expression of a wide range of genes, ATF4 is involved in many pathophysiological processes beyond ER stress, including redox balance, cell cycle, cell differentiation, autophagy, apoptosis, ferroptosis, amino acid uptake, glucose and fatty acid metabolism, *etc*., thus playing pivotal roles in a variety of diseases like cancer, neurodegenerative disease, metabolic disease, *etc*. (Tang et al., 2024; Xiao et al., 2023; He et al., 2023; Wei, Zhu & Liu, 2015; Ryan et al., 2021). For instance, in major urinary protein (MUP)-urokinase-type plasminogen activator (uPA) mice which were prone to ER stress, hepatocyte-derived ATF4 suppressed the progression of diethylnitrosamine- and high fat diet-induced hepatocellular carcinoma through upregulating the expression of solute carrier family 7a member 11 (SLC7A11), which was necessary for glutathione synthesis and protective against ferroptosis (He et al., 2023). As depicted in another study, ATF4 was robustly activated in the cellular models of Parkinson’s disease established by treatment of neurotoxins (Demmings et al., 2021). Furthermore, ATF4 deficiency inhibited neuronal apoptosis by transcriptionally downregulating the expression of pro-apoptotic factors CCAAT-enhancer binding protein homologous protein (CHOP), tribbles pseudokinase 3 (TRIB3) and p53 upregulated modulator of apoptosis (PUMA) (Demmings et al., 2021). These findings indicate that ATF4 plays dichotomous roles in different situations.

**Figure 2:**
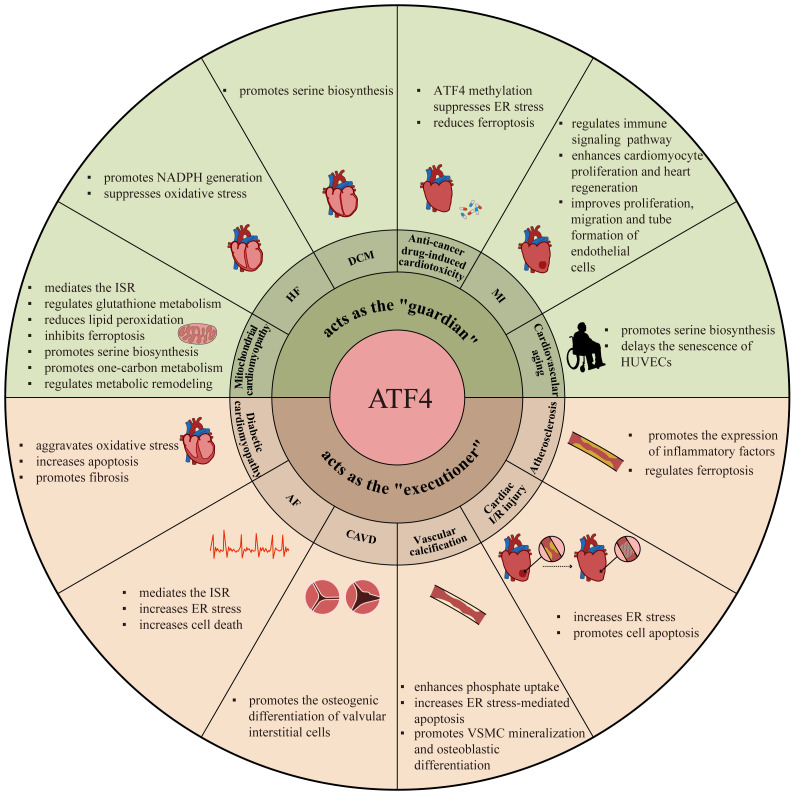
The dual roles and mechanisms of ATF4 in cardiovascular diseases. The involvement of ATF4 in various cardiovascular diseases, including coronary heart disease, cardiomyopathy, atrial fibrillation (AF), calcific aortic valve disease (CAVD), vascular calcification, heart failure (HF) and cardiovascular aging, and also highlights the relevant pathological processes regulated by ATF4 in these diseases. MI, myocardial infarction; I/R, ischemia/reperfusion; ER, endoplasmic reticulum; DCM, dilated cardiomyopathy; ISR, integrated stress response; NADPH, reduced nicotinamide adenine dinucleotide phosphate; HUVEC, human umbilical vein endothelial cell; VSMC, vascular smooth muscle cell.

Recently, ATF4 has become an increasingly prominent topic of investigations on cardiovascular diseases, and accumulating evidence has unveiled that the role of ATF4 in cardiovascular disorders is also dual-sided (Ahola et al., 2022; Wang et al., 2022; Zhu et al., 2023; Freundt et al., 2018). It acts as a protector maintaining cardiovascular homeostasis under certain stresses, yet becomes a driver of disease progression in specific pathological contexts (Wang et al., 2022; Freundt et al., 2018). This contradictory nature positions ATF4 as a pivotal molecule for understanding cardiovascular disease mechanisms and developing personalized targeted therapies. However, there remains a lack of systematic synthesis regarding the dichotomous roles of ATF4 in this field. Therefore, based on the cutting-edge evidence, this review aims to provide an overview of the conflicting roles of ATF4 in the pathogenesis of various cardiovascular diseases and also highlights the potential regulatory mechanisms (Fig. 2, Table 1). A comprehensive understanding of the dual effects and mechanisms of ATF4 in cardiovascular disorders holds promise for the development of personalized prevention and therapeutic avenues.

**Table 1:** The roles and mechanisms of ATF4 in cardiovascular diseases. This table lists the cardiovascular conditions, ATF4 expression patterns, downstream targets, functional roles, model systems and references, offering a comprehensive overview of the roles and mechanisms of ATF4 in cardiovascular diseases.

**Disease**	**Expression**	**Effect on downstream targets**	**Functional outcome**	**Cardiovascular significance**	**Model system**	**Reference**
Atherosclerosis	↑	↑ IL-8, IL-6, MCP-1, NF-κB pathway	↑ Endothelial inflammation	Aggravated	Endothelial cells *in vitro*	Gargalovic et al. (2006) and Gong et al. (2021)
Myocardial infarction	↑	↑ Genes related to cell division, such as *Knl1*	↑ Cardiomyocyte proliferation and heart regeneration	Protected	Mouse model	Gao et al. (2023)
		↑ PI3K/AKT signaling	↑ Angiogenesis	Protected	Mouse model	He et al. (2025)
Cardiac I/R injury	↑	↑ CHOP	↑ ER stress and apoptosis	Aggravated	AC16 cells and mouse models	Wang et al. (2021b) and Moulin et al. (2020)
Dilated cardiomyopathy		↑ PHGDH, TRIB3	↑ Serine biosynthesis	Protected	*In vitro* model using iPSC-CMs	Perea-Gil et al. (2022)
Diabetic cardiomyopathy	↑	↑ Smurf2	↑ Oxidative stress, cardiomyocyte apoptosis, cardiac fibrosis	Aggravated	Mouse model	Li et al. (2023)
Mitochondrial cardiomyopathy	↑		↓ ferroptosis	Protected	Mouse model	Ahola et al. (2022)
			↑ Metabolic shift from fatty acid oxidation to glucose metabolism			Kutschka et al. (2023)
Anticancer drug-induced cardiotoxicity	↑	↑ SLC7A11	↓ Ferroptosis	Protected	Mouse model	Jiang et al. (2022)
Atrial fibrillation	↑	↑ Genes related with amino acid biosynthesis, ER stress, cell death	↑ ER stress and cell death	Aggravated	HL-1 cells with rapid field stimulation	Freundt et al. (2018)
Calcific aortic valve disease	↑		↑ Osteogenic differentiation of valvular interstitial cells	Aggravated	Mouse model	Zhu et al. (2023), Li et al. (2020b) and Cai et al. (2013)
Heart failure	↑	↑ MTHFD2, G6PD, PHGDH, PSAT1	↓ Oxidative stress	Protected	Pressure overload-induced HF in mice	Wang et al. (2022)
Vascular calcification		↑ PiT1 and PiT2	↑ Phosphate uptake and apoptosis	Aggravated	*In vitro* and *in vivo*	Masuda et al. (2012) and Masuda et al. (2016)
Cardiovascular aging	↓	↑ PHGDH	↑ Serine biosynthesis	Protected	*In vitro* and *in vivo*	Wu et al. (2023)

**Notes.**

ATF4 activating transcription factor 4 IL interleukin MCP-1 monocyte chemoattractant protein 1 NF-κB nuclear factor kappa B PI3K phosphatidylinositol-3 kinase AKT protein kinase B I/R ischemia/reperfusion CHOP CCAAT-enhancer binding protein homologous protein ER endoplasmic reticulum PHGDH phosphoglycerate dehydrogenase TRIB3 tribbles pseudokinase 3 iPSC-CM induced pluripotent stem cell-derived cardiomyocyte Smurf2 Smad ubiquitin regulatory factor 2 SLC7A11 solute carrier family 7a member 11 MTHFD2 methylenetetrahydrofolate dehydrogenase 2 G6PD glucose-6-phosphate dehydrogenase PSAT1 phosphoserine aminotransferase 1

This review provides in-depth insights into the functional paradoxes and clinical translational potential of ATF4 in cardiovascular pathologies, which is conducive to promoting interdisciplinary cooperation to address the complexity of cardiovascular disease treatment. For academic researchers, this review fills in the gaps in the mechanistic understanding and points out potential directions for cross-disciplinary innovation. For clinicians and industry professionals, this review provides theoretical support for developing targeted therapy and accelerating the clinical translation. For educators, this review also serves as an educating material. Hence, this review is intended for readers across disciplines who are interested in ATF4, cellular stress response and cardiovascular diseases.

### Survey methodology

**Figure 3:**
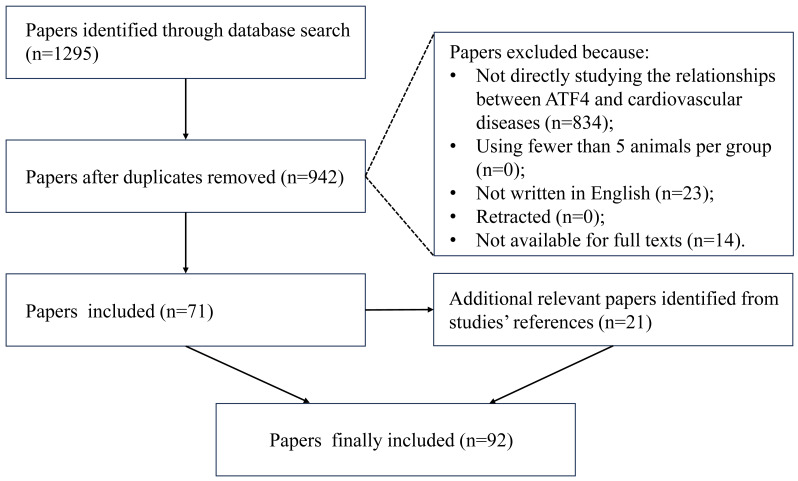
The flow diagram of literature screening.

Literature search was conducted in PubMed and Google Scholar by using a combination of keywords as follows: “activating transcription factor 4″OR “ATF4″, “integrated stress response” OR “ISR”, “cardiovascular disease” OR “heart disease” OR “coronary heart disease” OR “cardiomyopathy” OR “heart failure” OR “cardiovascular aging” OR “atrial fibrillation” OR “calcific aortic valvular disease” OR “vascular calcification”. We searched all relevant literature before September 2025. The selection process is presented in Fig. 3. After removing duplicate records, the remaining articles were screened based on the titles and abstracts. Then the full texts of potentially eligible studies were thoroughly assessed. We prioritized original research articles that directly investigated the role of ATF4 or the ISR in cardiovascular pathophysiology. To ensure the quality and reliability of the included studies, we applied the following considerations during selection:

1. Review articles were not included as primary sources of mechanistic evidence but were consulted for introducing background.
2. Animal studies using fewer than five animals per experimental group were excluded.
3. Non-English articles and retracted publications were excluded.
4. Articles that can not been accessed were excluded.

To ensure the comprehensiveness and objectivity of the screen results, Yaping Wang and Jie Yuan were responsible for searching the databases independently, then Feifan Wang and Hong Ma were in charge of evaluating the search results.

### Coronary heart disease

#### Atherosclerosis

With dyslipidemia as the major pathological basis, atherosclerosis constitutes the major cause of coronary heart disease (Gresham & Howard, 1960; Elias-Smale et al., 2011). In addition to dyslipidaemia, inflammation is also crucially involved in driving atherosclerosis (Libby, 2021). Previous research has revealed that ATF4 expression was elevated in human atherosclerotic lesions (Gargalovic et al., 2006; Sobolev et al., 2011). *In vitro*, ATF4 silencing significantly inhibited the expression of certain inflammatory factors, such as interleukin (IL)-8, IL-6 and monocyte chemoattractant protein 1 (MCP-1), in oxidized 1-palmitoyl-2-arachidonyl-sn-3-glycero-phosphorylcholine (oxPAPC)-treated human aortic endothelial cells (Gargalovic et al., 2006). Consistent with this finding, ATF4 silencing also attenuated inflammation in human umbilical vein endothelial cells (HUVECs) triggered by lysophosphatidycholine, the major component of oxidized low-density lipoprotein, through inhibiting the nuclear factor kappa B (NF-κB) pathway (Gong et al., 2021). These findings have underscored a potential role of ATF4 in controlling inflammatory response in atherosclerosis. Besides, ferroptosis also plays a crucial role in the pathogenesis of atherosclerosis (Wang et al., 2021a). Characterized by lipid peroxidation, ferroptosis is an iron-dependent programmed cell death distinct from other forms of cell death including apoptosis, necrosis and autophagy (Jiang, Stockwell & Conrad, 2021). Studies have indicated dual roles of ATF4 in regulating ferroptosis in different conditions, but its exact effect on ferroptosis in atherosclerosis still remains to be elucidated (Wang et al., 2021a).

#### Myocardial infarction (MI)

Myocardial infarction (MI) is the severe form of coronary heart disease that may result in ventricular remodeling, heart failure (HF), malignant arrhythmia, cardiac rupture and even death (Mehta et al., 2016). The bioinformatics analysis showed that ATF4 expression was increased and closely associated with several immune signaling pathways in the left ventricles of mice subjected to acute MI (Zhang et al., 2014). Besides, ATF4 is also involved in cardiomyocyte proliferation and heart regeneration (Gao et al., 2023). Over the past few decades, it has been believed that the adult mammalian heart almost loses the regeneration capacity so that it fails to produce enough cardiomyocytes to replace the infarcted myocardium (Zhang, Mignone & MacLellan, 2015). Intriguingly, emerging studies have indicated that promoting the proliferation of endogenous cardiomyocytes in damaged myocardial tissues can facilitate cardiac regeneration and prevent progression to HF (Garbern & Lee, 2022). A recent study showed that cardiac-specific reduction of mitochondrial ribosomal protein S5 (MRPS5), which was essential for mitochondrial protein translation, protected against MI by activating cardiomyocyte proliferation and heart regeneration in mice (Gao et al., 2023). Meanwhile, MRPS5 downregulation activated the ATF4 signaling pathway, while simultaneous knockout of MRPS5 and ATF4 inhibited cardiac regeneration and abolished the cardioprotective effects due to MRPS5 reduction (Gao et al., 2023). Further mechanistic investigation suggested that ATF4 enhanced cardiomyocyte proliferation and heart regeneration through transcriptionally upregulating the expression of genes related to cell division, such as *Knl1* (Gao et al., 2023). These findings support the key role of MRPS5/ATF4 in cardiomyocyte proliferation. ATF4 also contributes to endothelial cell function and neovascularization under hypoxic conditions (He et al., 2025). Overexpression of ATF4 improved the proliferation, migration and tube formation of hypoxic endothelial cells by modulating the PI3K/AKT signaling *in vitro*, while ATF4 inhibition led to impaired angiogenesis in endothelial cells. In line with these results, endothelial cell-specific overexpression of ATF4 by lentiviruses promoted post-MI neovascularization and mitigated cardiac dysfunction in mice (He et al., 2025). These results highlight ATF4 as a promising intervention target for treatment of MI.

#### Myocardial ischemia-reperfusion injury

The development of percutaneous coronary intervention and thrombolytic therapy makes it possible to promptly rescue the ischemic myocardium during MI, however, they also induce another kind of injury called ischemia-reperfusion (I/R) injury, which may influence cardiac prognosis (Xiang et al., 2024). According to a recent study, the N6-methyladenosine (m6A) modification of ATF4 mRNA was increased in cardiomyocytes during cardiac I/R injury, which was mediated by Wilms’ tumor 1-associating protein (WTAP), one of the components of m6A methyltransferase complex (Wang et al., 2021b). This modification led to enhanced mRNA stability and elevated protein level of ATF4, and further aggravated cardiomyocyte I/R injury by increasing ER stress and cell apoptosis (Wang et al., 2021b). With the characteristic of desaturation-reoxygenation cycling, chronic intermittent hypoxia due to sleep apnea can also cause cardiac I/R injury and cardiovascular comorbidities (Moulin et al., 2020). In mice subjected to chronic intermittent hypoxia during sleep and subsequent myocardial I/R injury, ATF4 expression was significantly increased in their hearts, and it promoted CHOP-mediated cardiomyocyte apoptosis through interacting with hypoxia-inducible factor 1α (HIF-1α) (Moulin et al., 2020). These results provide new insights into therapeutic approaches for alleviating cardiac I/R injury.

### Cardiomyopathy

#### Dilated cardiomyopathy

Characterized by left or bilateral ventricular enlargement with systolic dysfunction, dilated cardiomyopathy (DCM) is one of the major causes of HF as well as the most common reason for heart transplantation (Japp et al., 2016). It has poor prognosis with an estimated 5-year survival rate of approximately 50% (Xu et al., 2022). A recent study uncovered that ATF4-dependent serine biosynthesis could become a novel therapeutic target for DCM (Perea-Gil et al., 2022). By applying an *in vitro* DCM model recaptured by induced pluripotent stem cell-cardiomyocytes (iPSC-CMs) carrying the TNNT2 R183W mutation, this study showed that ATF4 was indispensable in mediating the beneficial effects of two small molecule kinase inhibitors, Gö 6976 and SB 203580, on rescuing the contractile function of cardiomyocytes (Perea-Gil et al., 2022). Mechanistically, the protective effect of ATF4 was achieved through promoting serine biosynthesis *via* targeting its downstream effectors phosphoglycerate dehydrogenase (PHGDH) and TRIB3 (Perea-Gil et al., 2022).

#### Diabetic cardiomyopathy

According to the latest consensus statement issued by the European Society of Cardiology in 2024, diabetic cardiomyopathy is newly defined as cardiac systolic and/or diastolic dysfunction in the presence of diabetes (Seferović et al., 2024). The pathogenesis of diabetic cardiomyopathy is complex because it is caused by the combined effects of diabetes and other concomitant factors, including hypertension, obesity, coronary heart disease, chronic kidney disease, *etc*. (Seferović et al., 2024) However, recent work sheds more light on the molecular mechanism of diabetic cardiomyopathy (Li et al., 2023). By analyzing the microarray data of iPSC-CMs exposed to glucose, endothelin-1 and cortisol, which mimicked the condition of diabetic cardiomyopathy *in vitro*, Li et al. (2020a) revealed that the differentially expressed genes between treated and untreated samples were mostly enriched in the metabolic process and cell cycle-related process, and further identified ATF4 as a novel prognostic biomarker of diabetic cardiomyopathy. Moreover, another *in vivo* study by Li et al. (2023) showed that ATF4 expression was significantly increased in the hearts of mice with diabetic cardiomyopathy, and deletion of ATF4 improved cardiac dysfunction through inhibiting oxidative stress, reducing cardiomyocyte apoptosis and alleviating cardiac fibrosis in diabetic mice. Further investigation indicated that ATF4 transcriptionally activated Smad ubiquitin regulatory factor 2 (Smurf2), and subsequently ubiquitinated and degradated homeodomain interacting protein kinase-2 (HIPK2), leading to the inactivation of the nuclear factor erythroid 2-related factor 2 (Nrf2)/heme oxygenase 1 (HO-1) pathway which classically functioned as protective mechanism against oxidative stress injury (Li et al., 2023). These results suggest that ATF4 exacerbates the progression of diabetic cardiomyopathy and offer new possible remedies for treating this disease.

#### Mitochondrial cardiomyopathy

Mitochondrial cardiomyopathy refers to cardiac involvement of mitochondrial disease, which is a hereditary disease caused by defects in mitochondrial energy metabolism (Yang et al., 2022). Previous research has indicated a strong link between ISR and mitochondrial dysfunction (Koncha et al., 2021; Khan et al., 2017). As a master effector of ISR, ATF4 has also been proved to play an important role in mitochondrial cardiomyopathy (Ahola et al., 2022; Huynh et al., 2023). In a mouse model of mitochondrial cardiomyopathy constructed by cardiac-specific deletion of Cox10, an assembly factor of the cytochrome c oxidase, activation of the Oma1-DAP3 binding cell death enhancer 1 (DELE1) axis could elicit ATF4-mediated ISR, which regulated glutathione metabolism, reduced lipid peroxidation and protected against ferroptosis, ultimately delayed the progression of cardiomyopathy (Ahola et al., 2022). In another study, ATF4 activation was also verified to mediate the protective effects of the mitochondrial protein DELE1 against mitochondrial cardiomyopathy established by cardiomyocyte-specific knockout of *Taz* in adult mice, as well as against fetal mitochondrial cardiomyopathy due to cardiomyocyte-restricted *Ptpmt1* deletion (Ahola et al., 2022; Huynh et al., 2023).

Specifically, Barth syndrome is an inherited mitochondrial disease mainly manifested as dilated cardiomyopathy, neutropenia and skeletal myopathy, which is caused by defect in the *Taz* gene (Sabbah, 2021). Unfortunately, there have been only symptomatic treatments for this fatal disease so far, eliciting urgent needs to further explore effective therapeutic targets. A metabolic shift from fatty acid oxidation to glucose metabolism has been discovered in the hearts of patients with Barth syndrome, but the underlying mechanism remains unclear (Chowdhury et al., 2023). Kutschka et al. (2023) demonstrated that activation of the eIF2α/ATF4 axis was crucially associated with the metabolic switch by using *Taz* knockdown mice which recaptured the phenotype of Barth syndrome. The cardiac transcriptome data showed that many of the differentially expressed genes between *Taz* knockdown and wildtype mice were transcriptionally regulated by ATF4 (Kutschka et al., 2023). On one hand, ATF4-induced transcriptomic changes promoted one-carbon metabolism, serine biosynthesis and cystine uptake to support the production of glutathione, an important antioxidant closely involved in the Kreb’s cycle and glucose metabolism (Kutschka et al., 2023). On the other hand, ATF4 facilitated the uptake and utilization of glutamate to fuel the Kreb’s cycle and energy production through anaplerotic replenishment (Kutschka et al., 2023). Together, these effects converged on driving cardiac metabolic remodeling to compensate for the mitochondrial functional deficits in Barth syndrome.

#### Anticancer drug-induced cardiotoxicity

Cancer therapy-related cardiotoxicity still remains a prominent clinical issue that may lead to severe and sometimes life-threatening cardiovascular events such as HF, myocarditis, arrhythmia and hypertension (Adhikari et al., 2021). However, there have been limited strategies to combat this serious side effect, except for liposomal formulation, dose reduction and drugs like dexrazoxane, antioxidants as well as HF medications (Wu et al., 2024b). Recent evidence has suggested that ATF4 may represent a potential target for alleviating this cardiotoxicity (Kim et al., 2022; Jiang et al., 2022). The work by Su Woo Kim’s team showed that protein arginine methyltransferase 1 (PRMT1) depletion aggravated oxidative stress, DNA damage and apoptosis in doxorubicin (Dox)-treated cardiomyocytes, which was contrary to PRMT1 overexpression (Kim et al., 2022). Interestingly, PRMT1 attenuated Dox-induced cardiotoxicity by promoting the methylation of ATF4 and suppressing ER stress (Kim et al., 2022). This study implies that ATF4 methylation may be a novel regulatory mechanism that protects cardiomyocytes from Dox-triggered death. Sorafenib, another broad-spectrum anti-tumor drug, can also result in severe cardiotoxicity (Liu et al., 2023). Jiang et al. (2022) found that sorafenib treatment caused obvious myocardial injury and cardiomyocyte hypertrophy in mice, which was reversed by the ferroptosis inhibitor Fer-1 and iron chelator desferrioxamine, indicating the involvement of ferroptosis in sorafenib-induced cardiomyopathy. In addition, the differentially expressed genes between cardiomyocytes treated with or without sorafenib were mostly enriched in the pathway of protein processing in ER, among which ATF4 was one of the most significantly upregulated (Jiang et al., 2022). Moreover, knockdown of ATF4 exacerbated sorafenib-induced cardiomyocyte toxicity both *in vivo* and *in vitro*, while ATF4 overexpression alleviated this phenomenon (Jiang et al., 2022). Mechanically, the protective effect of ATF4 was achieved through transcriptionally upregulating the expression of SLC7A11, which promoted glutathione biosynthesis, preserved redox balance and reduced ferroptosis (Jiang et al., 2022). These data suggest that ATF4 mitigates sorafenib-related cardiotoxicity through inhibiting ferroptosis.

### Heart failure

HF is the severe and terminal stage of various cardiovascular diseases. With 5-year mortality of nearly 50%, HF not only poses a major threat to patients’ health but also confers huge burden to the health-care system and social economy (Ziaeian & Fonarow, 2016). Thus, more efforts are needed to explore new potent intervention targets for HF. Redox homeostasis, maintained by generation and elimination of free radicals, is vital for cellular physiological activities (Sies, Mailloux & Jakob, 2024). Once the equilibrium state is disrupted, oxidative stress is induced. Oxidative stress is crucially associated with many kinds of diseases, including HF (Van der Pol et al., 2019; Tsutsui, Kinugawa & Matsushima, 2011). As a stress-induced transcription factor, ATF4 governs the expression of many oxidative stress-related genes, thus participating in the development of numerous diseases (Lange et al., 2008; Bagheri-Yarmand et al., 2019). Recent research revealed a protective role of ATF4 in HF *via* regulation of oxidative stress-related pathways (Wang et al., 2022). In pressure overload-induced HF, oxidative stress was induced partially due to decreased levels of the major antioxidants reduced nicotinamide adenine dinucleotide phosphate (NADPH) and glutathione (Wang et al., 2022). And ATF4 was proved to positively regulate the transcription of several enzymes such as methylenetetrahydrofolate dehydrogenase 2 (MTHFD2), glucose-6-phosphate dehydrogenase (G6PD), PHGDH, phosphoserine aminotransferase 1 (PSAT1), *etc*., which were critical for the generation of NADPH in the one-carbon metabolic pathway and pentose phosphate pathway (Wang et al., 2022). As expected, cardiomyocyte-specific deletion of ATF4 exacerbated pressure overload-induced cardiac dysfunction, fibrosis and apoptosis because of less production of NADPH and enhanced oxidative stress (Wang et al., 2022). This study provides evidence that targeting ATF4 may be a potential therapeutic strategy for the treatment of HF triggered by hemodynamic stress.

### Atrial fibrillation

As the most frequent arrhythmia managed in clinical practice, AF significantly increases the risk of stroke, dementia, HF and death (Sagris et al., 2021). Current treatments for atrial fibrillation (AF), such as catheter ablation, pharmacological or electrical cardioversion and anticoagulants, have unsatisfactory efficacy as well as serious side effects (Brundel et al., 2022). Therefore, it is necessary to further unravel the pathogenesis and find alternative therapeutic targets for AF. By using a rat model of MI, a recent study showed that administration of a novel small-molecule ISR inhibitor dramatically reduced macrophage infiltration, atrial fibrosis, cardiac dysfunction, and more importantly inhibited the vulnerability to AF induced by atrial burst rapid pacing (Zhang et al., 2021). These data indicate the close involvement of ISR in the development of AF. As the major effector of cellular ISR, ATF4 has also been implicated in the pathogenesis of AF (Freundt et al., 2018). In a cellular AF model constructed by treating the mouse atrial myocyte HL-1 cells with rapid field stimulation, ATF4 was significantly induced accompanied with the decrease of cell viability, and overexpression of ATF4 further reduced the viability of HL-1 cells through inducing the expression of numerous genes related with amino acid biosynthesis, ER stress and cell death (Freundt et al., 2018). Consistently, immunohistological analysis showed a higher number of ATF4 positive cardiomyocytes in the hearts of patients with AF compared to healthy controls (Freundt et al., 2018). These data unravel a prodeath role of ATF4 in the atrial remodeling of AF, suggesting that ATF4 inhibition may become a promising therapeutic method.

### Calcific aortic valve disease

Characterized by thickening, fibrosis and mineralization of the aortic valve leaflets, calcific aortic valve disease (CAVD) is one of the most prevalent valvular heart diseases and is associated with high morbidity and mortality in the elderly population (Moncla et al., 2023). However, there are currently no specific drugs that can effectively reverse or prevent the progression of CAVD (Peeters et al., 2018). Growing evidence has implicated a critical role of ATF4 in the development of CAVD (Zhu et al., 2023; Huang et al., 2023; Salim et al., 2019; Li et al., 2020b; Fu et al., 2019; Cai et al., 2013). The expression of ATF4 was significantly increased in murine CAVD model induced by high cholesterol diet, while knockdown of ATF4 inhibited the calcification of aortic valves *in vivo* (Zhu et al., 2023; Li et al., 2020b; Cai et al., 2013). The osteogenic differentiation of valvular interstitial cells (VICs) is considered one of the hallmarks in the occurrence of valve calcification (Huang et al., 2023). Indeed, the *in vitro* study showed that ATF4 knockdown also inhibited the osteogenic differentiation of VICs exposed to osteogenic induction medium (Zhu et al., 2023; Huang et al., 2023; Li et al., 2020b; Cai et al., 2013). These data suggest that ATF4 may serve as a potential therapeutic target for CAVD.

In addition to its direct regulatory effect, ATF4 is also involved in mediating the effects of other molecules on aortic valve calcification (Zhu et al., 2023; Huang et al., 2023). For instance, vascular smooth muscle cells-derived exosomes containing high levels of microRNA (miR)-129 and miR-342 inhibited the osteogenic differentiation of VICs and the development of CAVD, which was achieved by suppressing the eIF2α/ATF4 axis (Huang et al., 2023). As exhibited in another study, OxPAPC treatment promoted macrophage polarization towards M1 type, and co-culture with OxPAPC-preconditioned macrophages could enhance the osteogenic differentiation of VICs, which was abrogated by ATF4 knockdown (Zhu et al., 2023). This suggested that OxPAPC-polarized macrophages facilitated osteogenic differentiation of VICs and initiation of CAVD through upregulating ATF4 expression.

### Vascular calcification

Vascular calcification (VC), characterized by abnormal mineral deposition in the vasculature, is a major cardiovascular complication in patients with chronic kidney disease (CKD), diabetes, and atherosclerosis (Lee, Lee & Jeon, 2020). Far from a passive degenerative process, VC is now recognized as an active and regulated process analogous to osteogenesis, primarily mediated by the osteogenic transformation of vascular smooth muscle cells (VSMCs) (Masuda et al., 2012). A key driver of this transformation is the pro-osteogenic transcription factor ATF4 (Masuda et al., 2012). *In vitro*, ATF4 knockdown blunted stearate-induced VSMC mineralization and osteoblastic differentiation, whereas its overexpression was sufficient to drive calcification (Masuda et al., 2012). These findings were further supported by *in vivo* models (Masuda et al., 2016). Both global ATF4 haplodeficiency and smooth muscle cell (SMC)-specific ATF4 knockout reduced medial calcification in CKD mice (Masuda et al., 2016). Moreover, SMC-specific overexpression of ATF4 in mice triggered severe medial and atherosclerotic calcification even in the absence of CKD, underscoring that ATF4 activation in VSMCs is a pivotal event in the pathogenesis of VC (Masuda et al., 2016).

ATF4 promotes VC through multiple mechanisms. A key pathway is the transcriptional upregulation of type III sodium-dependent phosphate cotransporters PiT1 and PiT2 (Masuda et al., 2016). ATF4 forms a complex with C/EBPβ, and this heterodimer binds to specific response elements in the PiT1 and PiT2 genes, thereby increasing their expression and enhancing phosphate uptake—a critical step in VSMC calcification (Masuda et al., 2016). Additionally, ATF4 contributes to ER stress-mediated apoptosis, another key process of VC. Duan et al. (2013) showed that ATF4 was activated in calcified aortas and VSMCs, and its knockdown attenuated the expression of the pro-apoptotic factor CHOP, subsequently inhibiting apoptosis and calcification in VSMCs. This aligns with the *in vivo* data showing that ATF4 deficiency reduced TUNEL-positive cells and CHOP expression in calcified aortas (Masuda et al., 2016).

In summary, ATF4 acts as a central regulator of VC, suggesting that targeted inhibition of the ATF4 pathway could be a promising therapeutic strategy.

### Cardiovascular aging

Cardiovascular aging is a progressive process involving age-induced alterations in the structure and function of the cardiovascular system that may lead to increased risk of cardiovascular diseases (Paneni et al., 2017). Cellular senescence is one of the hallmarks of cardiovascular aging (Abdellatif et al., 2023). And changes in energy metabolism has been considered one of the main mechanisms underlying cellular senescence (Shmulevich & Krizhanovsky, 2021). According to a recent study, the transcriptome between replicative senescent and young HUVECs exhibited tremendous changes, especially for genes related to glycine, serine and threonine metabolism, among which the mRNA level of PHGDH changed most obviously (Wu et al., 2023). PHGDH is one of the rate-limiting enzymes in serine biosynthesis (Zhang et al., 2023). PHGDH knockdown accelerated while PHGDH overexpression delayed the senescence of HUVECs, and vascular endothelium-specific overexpression of PHGDH prevented dilated cardiomyopathy, improved cardiac function, promoted endothelial function and slowed down cellular senescence in aged mice (Wu et al., 2023). ATF4 is the transcription factor responsible for orchestrating the expression of PHGDH (Yoon et al., 2023). Consistent with the above results, ATF4 knockdown inhibited the expression of PHGDH, reduced intracellular serine levels, as well as accelerated cellular senescence in HUVECs, which was partially reversed by addition of serine (Wu et al., 2023). Taken together, this study supports that the ATF4/PHGDH axis may represent a promising target for delaying cellular senescence and age-related cardiovascular diseases.

### The context-dependent duality of ATF4: underlying mechanisms

The preceding sections illustrate a central paradox: ATF4 can function as either a guardian of cellular homeostasis or an executioner of cell death in the cardiovascular system. This functional duality is dictated by a complex interplay of specific cellular and molecular contexts. Understanding the mechanisms that govern this balance is crucial for developing targeted therapeutic strategies. First, the nature and duration of cellular stress is a critical determinant of ATF4’s functional outcome (Chen et al., 2025). Under transient and mild stress, ATF4 orchestrates a pro-survival program that promotes adaption and restores homeostasis. However, severe or prolonged stress leads to sustained ATF4 activation, which drives the expression of pro-apoptotic factors such as CHOP, ultimately tipping the balance toward cell death (Chen et al., 2025). Second, the functional outcome of ATF4 is also governed by its dimerization partners, which dictate the transcriptional selectivity and cellular fate (Pakos-Zebrucka et al., 2016). For instance, the ATF4-CHOP heterodimer drives cell death, whereas its dimerization with ATF3 supports cellular adaption and recovery of homeostasis (Ohoka et al., 2005; Wang et al., 2009). Furthermore, ATF4 is subject to multiple post-translational modifications (PTMs) that regulate its stability, localization and transcriptional activity (Pakos-Zebrucka et al., 2016). For example, phosphorylation by ribosomal protein S6 kinase α-2 (RSK2) enhances the stability and transcriptional activity of ATF4, thereby amplifying either pro-survival or pro-death signaling depending on the context (Yang et al., 2004). Conversely, deacetylation by SIRT1 can suppress its transcriptional activity (Woo et al., 2013).

## Conclusions

Despite remarkable advances in the development of preventive and therapeutic strategies, cardiovascular disease still remains the leading cause of mortality and excessive medical costs worldwide (Chong et al., 2025). Thus it is of great significance to focus on research on the pathogenesis of cardiovascular pathologies and explore novel effective interventions to reduce the disease burden and improve global health. This review provides an insight into the dichotomous roles and corresponding mechanisms of ATF4 in different kinds of cardiovascular disorders. Namely, ATF4 exhibits both protective and detrimental effects in a context-dependent manner. The contrasting roles emphasize the need for context-specific modulation of ATF4 activity, paving the way for ATF4-targeted precision therapy in clinical practice. However, our understanding of ATF4 signaling in different cardiac cell types remains limited and represents an important direction for future research. In particular, the cell type-specific functions of ATF4 in non-cardiomyocytes, including endothelial cells, fibroblasts and immune cells within the heart are still poorly defined and warrant systematic investigation. Further studies are required to explore the differential regulatory networks of ATF4 across these cell types and their interactions with the microenvironment. Besides, the mechanism of functional transformation of ATF4 during different stages of cardiovascular diseases also remains to be elucidated.

The compelling evidence of ATF4’s dual roles in cardiovascular pathologies underscores its potential as a therapeutic target, yet translating this into clinical practice faces several key challenges. First, most transcription factors lack well-defined binding pockets for small-molecule inhibitors, making it challenging for direct pharmacological inhibition (Henley & Koehler, 2021). This can be addressed through several alternative strategies: (1) developing small molecules that disrupt the protein-protein interactions between ATF4 and its binding partners; (2) Regulating the stability and activity of ATF4 through post-translational modifications; (3) employing gene-silencing approaches using siRNA to target ATF4 at the mRNA level. Second, the ubiquitous expression and pleiotropic functions of ATF4 suggest that systemic inhibition could raise the risk of unintended side effects. Thus, it is needed to develop effective and safe delivery systems that can transport the therapeutic agents specifically to the cardiovascular system. While lipid nanoparticles and viral vectors are promising candidates, the potential issues related to their immunogenicity, durability and tissue specificity remain to be optimized (Raguram, Banskota & Liu, 2022). Third, the context-dependent duality of ATF4 demands cell-type specific targeting strategies. This could be achieved by coupling therapeutic agents to ligands or antibodies that recognize unique surface markers on pathogenic cell populations (Raguram, Banskota & Liu, 2022). Additionally, current data are mainly derived from preclinical experiments, and there exists several limitations of the experimental models used. For instance, the HL-1 cell line does not fully recapitulate the metabolic and electrophysiological properties of adult cardiomyocytes *in vivo*. Similarly, the iPSC-CMs often exhibit an immature phenotype, resembling fetal rather than adult cardiomyocytes. Moreover, while indispensable for *in vivo* studies, genetically modified mouse models have significant translational gaps from humans. Collectively, these models may not fully recapitulate the pathophysiological context of human cardiovascular diseases. Therefore, future clinical research is warranted to prove the efficacy and safety of ATF4-targeted strategies in patients.

Multidisciplinary cooperation may exert profound effects in promoting the development of therapeutic strategies targeting ATF4. For instance, by cooperating with the traditional Chinese medicine teams, we can screen the traditional drug components that can regulate ATF4 to protect against cardiovascular diseases. Combined with the knowledge of materials science, nano-carriers that target cardiovascular system can be designed for the delivery of ATF4 inhibitors or siRNA, thereby reducing the systemic side effects. Moreover, machine learning and bioinformatics can be applied to study the interacting network of ATF4 with other proteins to further explore novel inhibitors.

In summary, as the core regulator of cellular stress, ATF4 plays essential context-specific roles in cardiovascular diseases, and targeting ATF4 may represent novel therapeutic approaches for cardiovascular pathologies. More efforts are needed to further clarify the molecular mechanisms and promote clinical translation, ultimately achieving the leap from stress sentinel to intervention target for cardiovascular diseases.

## Additional Information and Declarations

### Data Availability

The following information was supplied regarding data availability: Raw data was not generated in this literature review.

## References

[ref-1] Abdellatif M, Rainer PP, Sedej S, Kroemer G (2023). Hallmarks of cardiovascular ageing. Nature Reviews Cardiology.

[ref-2] Adams CM, Ebert SM, Dyle MC (2017). Role of ATF4 in skeletal muscle atrophy. Current Opinion in Clinical Nutrition & Metabolic Care.

[ref-3] Adhikari A, Asdaq SMB, Al Hawaj MA, Chakraborty M, Thapa G, Bhuyan NR, Imran M, Alshammari MK, Alshehri MM, Harshan AA, Alanazi A, Alhazmi BD, Sreeharsha N (2021). Anticancer drug-induced cardiotoxicity: insights and pharmacogenetics. Pharmaceuticals.

[ref-4] Ahola S, Rivera Mejías P, Hermans S, Chandragiri S, Giavalisco P, Nolte H, Langer T (2022). OMA1-mediated integrated stress response protects against ferroptosis in mitochondrial cardiomyopathy. Cell Metabolism.

[ref-5] Ameri K, Harris AL (2008). Activating transcription factor 4. International Journal of Biochemistry and Cell Biology.

[ref-6] Bagheri-Yarmand R, Sinha KM, Li L, Lu Y, Cote GJ, Sherman SI, Gagel RF (2019). Combinations of tyrosine kinase inhibitor and ERAD inhibitor promote oxidative stress-induced apoptosis through ATF4 and KLF9 in medullary thyroid cancer. Molecular Cancer Research.

[ref-7] Brundel B, Ai X, Hills MT, Kuipers MF, Lip GYH, De Groot NMS (2022). Atrial fibrillation. Nature Reviews Disease Primers.

[ref-8] Cai Z, Li F, Gong W, Liu W, Duan Q, Chen C, Ni L, Xia Y, Cianflone K, Dong N, Wang DW (2013). Endoplasmic reticulum stress participates in aortic valve calcification in hypercholesterolemic animals. Arteriosclerosis, Thrombosis, and Vascular Biology.

[ref-9] Chen CW, Papadopoli D, Szkop KJ, Guan BJ, Alzahrani M, Wu J, Jobava R, Asraf MM, Krokowski D, Vourekas A, Merrick WC, Komar AA, Koromilas AE, Gorospe M, Payea MJ, Wang F, Clayton BLL, Tesar PJ, Schaffer A, Miron A, Bederman I, Jankowsky E, Vogel C, Valášek LS, Dinman JD, Zhang Y, Tirosh B, Larsson O, Topisirovic I, Hatzoglou M (2025). Plasticity of the mammalian integrated stress response. Nature.

[ref-10] Chong B, Jayabaskaran J, Jauhari SM, Chan SP, Goh R, Kueh MTW, Li H, Chin YH, Kong G, Anand VV, Wang JW, Muthiah M, Jain V, Mehta A, Lim SL, Foo R, Figtree GA, Nicholls SJ, Mamas MA, Januzzi JL, Chew NWS, Richards AM, Chan MY (2025). Global burden of cardiovascular diseases: projections from 2025 to 2050. European Journal of Preventive Cardiology.

[ref-11] Chowdhury A, Boshnakovska A, Aich A, Methi A, Vergel Leon AM, Silbern I, Lüchtenborg C, Cyganek L, Prochazka J, Sedlacek R, Lindovsky J, Wachs D, Nichtova Z, Zudova D, Koubkova G, Fischer A, Urlaub H, Brügger B, Katschinski DM, Dudek J, Rehling P (2023). Metabolic switch from fatty acid oxidation to glycolysis in knock-in mouse model of Barth syndrome. EMBO Molecular Medicine.

[ref-12] Demmings MD, Tennyson EC, Petroff GN, Tarnowski-Garner HE, Cregan SP (2021). Activating transcription factor-4 promotes neuronal death induced by Parkinson’s disease neurotoxins and α-synuclein aggregates. Cell Death and Differentiation.

[ref-13] Duan XH, Chang JR, Zhang J, Zhang BH, Li YL, Teng X, Zhu Y, Du J, Tang CS, Qi YF (2013). Activating transcription factor 4 is involved in endoplasmic reticulum stress-mediated apoptosis contributing to vascular calcification. Apoptosis.

[ref-14] Ebert SM, Rasmussen BB, Judge AR, Judge SM, Larsson L, Wek RC, Anthony TG, Marcotte GR, Miller MJ, Yorek MA, Vella A, Volpi E, Stern JI, Strub MD, Ryan Z, Talley JJ, Adams CM (2022). Biology of activating transcription factor 4 (ATF4) and its role in skeletal muscle atrophy. Journal of Nutrition.

[ref-15] Elias-Smale SE, Wieberdink RG, Odink AE, Hofman A, Hunink MG, Koudstaal PJ, Krestin GP, Breteler MM, Van der Lugt A, Witteman JC (2011). Burden of atherosclerosis improves the prediction of coronary heart disease but not cerebrovascular events: the Rotterdam study. European Heart Journal.

[ref-16] Freundt JK, Frommeyer G, Wötzel F, Huge A, Hoffmeier A, Martens S, Eckardt L, Lange PS (2018). The transcription factor ATF4 promotes expression of cell stress genes and cardiomyocyte death in a cellular model of atrial fibrillation. BioMed Research International.

[ref-17] Fu Z, Li F, Jia L, Su S, Wang Y, Cai Z, Xiang M (2019). Histone deacetylase 6 reduction promotes aortic valve calcification *via* an endoplasmic reticulum stress-mediated osteogenic pathway. Journal of Thoracic and Cardiovascular Surgery.

[ref-18] Gao F, Liang T, Lu YW, Pu L, Fu X, Dong X, Hong T, Zhang F, Liu N, Zhou Y, Wang H, Liang P, Guo Y, Yu H, Zhu W, Hu X, Chen H, Zhou B, Pu WT, Mably JD, Wang J, Wang DZ, Chen J (2023). Reduced mitochondrial protein translation promotes cardiomyocyte proliferation and heart regeneration. Circulation.

[ref-19] Garbern JC, Lee RT (2022). Heart regeneration: 20 years of progress and renewed optimism. Developmental Cell.

[ref-20] Gargalovic PS, Gharavi NM, Clark MJ, Pagnon J, Yang WP, He A, Truong A, Baruch-Oren T, Berliner JA, Kirchgessner TG, Lusis AJ (2006). The unfolded protein response is an important regulator of inflammatory genes in endothelial cells. Arteriosclerosis, Thrombosis, and Vascular Biology.

[ref-21] GBD 2017 Causes of Death Collaborators (2018). Global, regional, and national age-sex-specific mortality for 282 causes of death in 195 countries and territories, 1980–2017: a systematic analysis for the global burden of disease study 2017. Lancet.

[ref-22] Gong Y, Li Q, Ma Z, Jin T, Lin J, Lv Q, Wang M, Fu G, Xu S (2021). Downregulation of activating transcription factor 4 attenuates lysophosphatidycholine-induced inflammation *via* the NF-κB pathway. European Journal of Pharmacology.

[ref-23] Gresham GA, Howard AN (1960). Atherosclerosis and coronary heart-disease. Lancet.

[ref-24] He P, Zeng W, Li J, Zhang Y, Zhao R, Liu W, Zhao Y, Liu Z, Shen C, Chen W, Wang Y, Shi B (2025). ATF4 regulates PI3K/AKT signaling axis to promote angiogenesis after myocardial infarction. In Vitro Cellular & Developmental Biology-Animal.

[ref-25] He F, Zhang P, Liu J, Wang R, Kaufman RJ, Yaden BC, Karin M (2023). ATF4 suppresses hepatocarcinogenesis by inducing SLC7A11 (xCT) to block stress-related ferroptosis. Journal of Hepatology.

[ref-26] Henley MJ, Koehler AN (2021). Advances in targeting ‘undruggable’ transcription factors with small molecules. Nature Reviews Drug Discovery.

[ref-27] Huang C, Han X, Yang L, Song W, Zhang H, Zhu X, Huang G, Xu J (2023). Exosomal miR-129 and miR-342 derived from intermittent hypoxia-stimulated vascular smooth muscle cells inhibit the eIF2α/ATF4 axis from preventing calcified aortic valvular disease. Journal of Cell Communication and Signaling.

[ref-28] Huynh H, Zhu S, Lee S, Bao Y, Pang J, Nguyen A, Gu Y, Chen C, Ouyang K, Evans SM, Fang X (2023). DELE1 is protective for mitochondrial cardiomyopathy. Journal of Molecular and Cellular Cardiology.

[ref-29] Japp AG, Gulati A, Cook SA, Cowie MR, Prasad SK (2016). The diagnosis and evaluation of dilated cardiomyopathy. Journal of the American College of Cardiology.

[ref-30] Jiang X, Stockwell BR, Conrad M (2021). Ferroptosis: mechanisms, biology and role in disease. Nature Reviews Molecular Cell Biology.

[ref-31] Jiang H, Wang C, Zhang A, Li Y, Li J, Li Z, Yang X, Hou Y (2022). ATF4 protects against sorafenib-induced cardiotoxicity by suppressing ferroptosis. Biomedicine and Pharmacotherapy.

[ref-32] Khan NA, Nikkanen J, Yatsuga S, Jackson C, Wang L, Pradhan S, Kivelä R, Pessia A, Velagapudi V, Suomalainen A (2017). mTORC1 regulates mitochondrial integrated stress response and mitochondrial myopathy progression. Cell Metabolism.

[ref-33] Kim SW, Ahn BY, Tran TTV, Pyun JH, Kang JS, Leem YE (2022). PRMT1 suppresses doxorubicin-induced cardiotoxicity by inhibiting endoplasmic reticulum stress. Cellular Signalling.

[ref-34] Koncha RR, Ramachandran G, Sepuri NBV, Ramaiah KVA (2021). CCCP-induced mitochondrial dysfunction—characterization and analysis of integrated stress response to cellular signaling and homeostasis. The FEBS Journal.

[ref-35] Kutschka I, Bertero E, Wasmus C, Xiao K, Yang L, Chen X, Oshima Y, Fischer M, Erk M, Arslan B, Alhasan L, Grosser D, Ermer KJ, Nickel A, Kohlhaas M, Eberl H, Rebs S, Streckfuss-Bömeke K, Schmitz W, Rehling P, Thum T, Higuchi T, Rabinowitz J, Maack C, Dudek J (2023). Activation of the integrated stress response rewires cardiac metabolism in Barth syndrome. Basic Research in Cardiology.

[ref-36] Lange PS, Chavez JC, Pinto JT, Coppola G, Sun CW, Townes TM, Geschwind DH, Ratan RR (2008). ATF4 is an oxidative stress-inducible, prodeath transcription factor in neurons *in vitro* and *in vivo*. Journal of Experimetnal Medicine.

[ref-37] Lee SJ, Lee IK, Jeon JH (2020). Vascular calcification-new insights into its mechanism. International Journal of Molecular Sciences.

[ref-38] Li N, Bai Y, Zhou G, Ma Y, Tan M, Qiao F, Li X, Shen M, Song X, Zhao X, Liu X, Xu Z (2020b). miR-214 attenuates aortic valve calcification by regulating osteogenic differentiation of valvular interstitial cells. Molecular Therapy—Nucleic Acids.

[ref-39] Li Y, He Q, He CY, Cai C, Chen Z, Duan JZ (2023). Activating transcription factor 4 drives the progression of diabetic cardiac fibrosis. ESC Heart Fail.

[ref-40] Li H, Li X, Guo J, Wu G, Dong C, Pang Y, Gao S, Wang Y (2020a). Identification of biomarkers and mechanisms of diabetic cardiomyopathy using microarray data. The Journal of Cardiology.

[ref-41] Libby P (2021). The changing landscape of atherosclerosis. Nature.

[ref-42] Liu S, Yue S, Guo Y, Han JY, Wang H (2023). Sorafenib induces cardiotoxicity through RBM20-mediated alternative splicing of sarcomeric and mitochondrial genes. Pharmacological Research.

[ref-43] Lu HJ, Koju N, Sheng R (2024). Mammalian integrated stress responses in stressed organelles and their functions. Acta Pharmacologica Sinica.

[ref-44] Masuda M, Miyazaki-Anzai S, Keenan AL, Shiozaki Y, Okamura K, Chick WS, Williams K, Zhao X, Rahman SM, Tintut Y, Adams CM, Miyazaki M (2016). Activating transcription factor-4 promotes mineralization in vascular smooth muscle cells. JCI Insight.

[ref-45] Masuda M, Ting TC, Levi M, Saunders SJ, Miyazaki-Anzai S, Miyazaki M (2012). Activating transcription factor 4 regulates stearate-induced vascular calcification. Journal of Lipid Research.

[ref-46] Mehta LS, Beckie TM, DeVon HA, Grines CL, Krumholz HM, Johnson MN, Lindley KJ, Vaccarino V, Wang TY, Watson KE, Wenger NK (2016). Acute myocardial infarction in women: a scientific statement from the American Heart Association. Circulation.

[ref-47] Moncla LM, Briend M, Bossé Y, Mathieu P (2023). Calcific aortic valve disease: mechanisms, prevention and treatment. Nature Reviews Cardiology.

[ref-48] Moulin S, Thomas A, Arnaud C, Arzt M, Wagner S, Maier LS, Pépin JL, Godin-Ribuot D, Gaucher J, Belaidi E (2020). Cooperation between hypoxia-inducible factor 1α and activating transcription factor 4 in sleep apnea-mediated myocardial injury. Canadian Journal of Cardiology.

[ref-49] Neill G, Masson GR (2023). A stay of execution: ATF4 regulation and potential outcomes for the integrated stress response. Frontiers in Molecular Neuroscience.

[ref-50] Ohoka N, Yoshii S, Hattori T, Onozaki K, Hayashi H (2005). TRB3, a novel ER stress-inducible gene, is induced *via* ATF4-CHOP pathway and is involved in cell death. The EMBO Journal.

[ref-51] Pakos-Zebrucka K, Koryga I, Mnich K, Ljujic M, Samali A, Gorman AM (2016). The integrated stress response. EMBO Reports.

[ref-52] Paneni F, Diaz Cañestro C, Libby P, Lüscher TF, Camici GG (2017). The aging cardiovascular system: understanding it at the cellular and clinical levels. Journal of the American College of Cardiology.

[ref-53] Peeters F, Meex SJR, Dweck MR, Aikawa E, Crijns H, Schurgers LJ, Kietselaer B (2018). Calcific aortic valve stenosis: hard disease in the heart: a biomolecular approach towards diagnosis and treatment. European Heart Journal.

[ref-54] Perea-Gil I, Seeger T, Bruyneel AAN, Termglinchan V, Monte E, Lim EW, Vadgama N, Furihata T, Gavidia AA, Arthur Ataam J, Bharucha N, Martinez-Amador N, Ameen M, Nair P, Serrano R, Kaur B, Feyen DAM, Diecke S, Snyder MP, Metallo CM, Mercola M, Karakikes I (2022). Serine biosynthesis as a novel therapeutic target for dilated cardiomyopathy. European Heart Journal.

[ref-55] Raguram A, Banskota S, Liu DR (2022). Therapeutic *in vivo* delivery of gene editing agents. Cell.

[ref-56] Ranea-Robles P, Pavlova NN, Bender A, Pereyra AS, Ellis JM, Stauffer B, Yu C, Thompson CB, Argmann C, Puchowicz M, Houten SM (2022). A mitochondrial long-chain fatty acid oxidation defect leads to transfer RNA uncharging and activation of the integrated stress response in the mouse heart. Cardiovascular Research.

[ref-57] Ryan DG, Yang M, Prag HA, Blanco GR, Nikitopoulou E, Segarra-Mondejar M, Powell CA, Young T, Burger N, Miljkovic JL, Minczuk M, Murphy MP, Von Kriegsheim A, Frezza C (2021). Disruption of the TCA cycle reveals an ATF4-dependent integration of redox and amino acid metabolism. Elife.

[ref-58] Ryoo HD (2024). The integrated stress response in metabolic adaptation. Journal of Biological Chemistry.

[ref-59] Sabbah HN (2021). Barth syndrome cardiomyopathy: targeting the mitochondria with elamipretide. Heart Failure Reviews.

[ref-60] Sagris M, Vardas EP, Theofilis P, Antonopoulos AS, Oikonomou E, Tousoulis D (2021). Atrial fibrillation: pathogenesis, predisposing factors, and genetics. International Journal of Molecular Sciences.

[ref-61] Salim MT, Esmerats JF, Arjunon S, Villa-Roel N, Nerem RM, Jo H, Yoganathan AP (2019). miR-214 is stretch-sensitive in aortic valve and inhibits aortic valve calcification. Annals of Biomedical Engineering.

[ref-62] Seferović PM, Paulus WJ, Rosano G, Polovina M, Petrie MC, Jhund PS, Tschöpe C, Sattar N, Piepoli M, Papp Z, Standl E, Mamas MA, Valensi P, Linhart A, Lalić N, Ceriello A, Döhner W, Ristić A, Milinković I, Seferović J, Cosentino F, Metra M, Coats AJS (2024). Diabetic myocardial disorder. A clinical consensus statement of the Heart Failure Association of the ESC and the ESC working group on myocardial & pericardial diseases. European Journal of Heart Failure.

[ref-63] Shmulevich R, Krizhanovsky V (2021). Cell senescence, DNA damage, and metabolism. Antioxid Redox Signal.

[ref-64] Sies H, Mailloux RJ, Jakob U (2024). Fundamentals of redox regulation in biology. Nature Reviews Molecular Cell Biology.

[ref-65] Sobolev VV, Starodubtseva NL, Piruzyan AL, Minnibaev MT, Sautin ME, Tumanov VP, Bruskin SA (2011). Comparative study of the expression of ATF-3 and ATF-4 genes in vessels involved into atherosclerosis process and in psoriatic skin. Bulletin of Experimental Biology and Medicine.

[ref-66] Soppert J, Lehrke M, Marx N, Jankowski J, Noels H (2020). Lipoproteins and lipids in cardiovascular disease: from mechanistic insights to therapeutic targeting. Advanced Drug Delivery Reviews.

[ref-67] Tang H, Kang R, Liu J, Tang D (2024). ATF4 in cellular stress, ferroptosis, and cancer. Archives of Toxicology.

[ref-68] Tian X, Zhang S, Zhou L, Seyhan AA, Hernandez Borrero L, Zhang Y, El-Deiry WS (2021). Targeting the integrated stress response in cancer therapy. Frontiers in Pharmacology.

[ref-69] Tsutsui H, Kinugawa S, Matsushima S (2011). Oxidative stress and heart failure. The American Journal of Physiology-Heart and Circulatory Physiology.

[ref-70] Van der Pol A, Van Gilst WH, Voors AA, Van der Meer P (2019). Treating oxidative stress in heart failure: past, present and future. European Journal of Heart Failure.

[ref-71] Wang Q, Mora-Jensen H, Weniger MA, Perez-Galan P, Wolford C, Hai T, Ron D, Chen W, Trenkle W, Wiestner A, Ye Y (2009). ERAD inhibitors integrate ER stress with an epigenetic mechanism to activate BH3-only protein NOXA in cancer cells. Proceedings of the National Academy of Sciences of the United States of America.

[ref-72] Wang X, Zhang G, Dasgupta S, Niewold EL, Li C, Li Q, Luo X, Tan L, Ferdous A, Lorenzi PL, Rothermel BA, Gillette TG, Adams CM, Scherer PE, Hill JA, Wang ZV (2022). ATF4 protects the heart from failure by antagonizing oxidative stress. Circulation Research.

[ref-73] Wang J, Zhang J, Ma Y, Zeng Y, Lu C, Yang F, Jiang N, Zhang X, Wang Y, Xu Y, Hou H, Jiang S, Zhuang S (2021b). WTAP promotes myocardial ischemia/reperfusion injury by increasing endoplasmic reticulum stress *via* regulating m(6)A modification of ATF4 mRNA. Aging.

[ref-74] Wang Y, Zhao Y, Ye T, Yang L, Shen Y, Li H (2021a). Ferroptosis signaling and regulators in atherosclerosis. Frontiers in Cell and Developmental Biology.

[ref-75] Wei N, Zhu LQ, Liu D (2015). ATF4: a novel potential therapeutic target for Alzheimer’s disease. Molecular Neurobiology.

[ref-76] Woo SR, Park JE, Kim YH, Ju YJ, Shin HJ, Joo HY, Park ER, Hong SH, Park GH, Lee KH (2013). SIRT1 suppresses activating transcription factor 4 (ATF4) expression in response to proteasome inhibition. Journal of Microbiology and Biotechnology.

[ref-77] Wu Y, Tang L, Huang H, Yu Q, Hu B, Wang G, Ge F, Yin T, Li S, Yu X (2023). Phosphoglycerate dehydrogenase activates PKM2 to phosphorylate histone H3T11 and attenuate cellular senescence. Nature Communications.

[ref-78] Wu L, Zhang Y, Wang G, Ren J (2024b). Molecular mechanisms and therapeutic targeting of ferroptosis in doxorubicin-induced cardiotoxicity. JACC: Basic to Translational Science.

[ref-79] Wu Y, Zhang H, Wang Y, Zhang Y, Hong Z, Wang D (2024a). Sephin1 enhances integrated stress response and autophagy to alleviate myocardial ischemia-reperfusion injury in mice. Biomedicine and Pharmacotherapy.

[ref-80] Xiang Q, Yi X, Zhu XH, Wei X, Jiang DS (2024). Regulated cell death in myocardial ischemia-reperfusion injury. Trends in Endocrinology & Metabolism.

[ref-81] Xiao Y, Xie X, Chen Z, Yin G, Kong W, Zhou J (2023). Advances in the roles of ATF4 in osteoporosis. Biomedicine and Pharmacotherapy.

[ref-82] Xu XR, Han MM, Yang YZ, Wang X, Hou DY, Meng XC, Wang H, Zhao WS, Zhang L, Xu L (2022). Fifteen-year mortality and prognostic factors in patients with dilated cardiomyopathy: persistent standardized application of drug therapy and strengthened management may bring about encouraging change in an aging society. Journal of Geriatric Cardiology.

[ref-83] Yan G, Han Z, Kwon Y, Jousma J, Nukala SB, Prosser BL, Du X, Pinho S, Ong SB, Lee WH, Ong SG (2024). Integrated stress response potentiates ponatinib-induced cardiotoxicity. Circulation Research.

[ref-84] Yang J, Chen S, Duan F, Wang X, Zhang X, Lian B, Kou M, Chiang Z, Li Z, Lian Q (2022). Mitochondrial cardiomyopathy: molecular epidemiology, diagnosis, models, and therapeutic management. Cell.

[ref-85] Yang X, Matsuda K, Bialek P, Jacquot S, Masuoka HC, Schinke T, Li L, Brancorsini S, Sassone-Corsi P, Townes TM, Hanauer A, Karsenty G (2004). ATF4 is a substrate of RSK2 and an essential regulator of osteoblast biology; implication for coffin-lowry syndrome. Cell.

[ref-86] Yoon BK, Kim H, Oh TG, Oh SK, Jo S, Kim M, Chun KH, Hwang N, Lee S, Jin S, Atkins AR, Yu RT, Downes M, Kim JW, Kim H, Evans RM, Cheong JH, Fang S (2023). PHGDH preserves one-carbon cycle to confer metabolic plasticity in chemoresistant gastric cancer during nutrient stress. Proceedings of the National Academy of Sciences of the United States of America.

[ref-87] Zhang D, Li AM, Hu G, Huang M, Yang F, Zhang L, Wellen KE, Xu X, Conn CS, Zou W, Kahn M, Rhoades SD, Weljie AM, Fuchs SY, Amankulor N, Yoshor D, Ye J, Koumenis C, Gong Y, Fan Y (2023). PHGDH-mediated endothelial metabolism drives glioblastoma resistance to chimeric antigen receptor T cell immunotherapy. Cell Metabolism.

[ref-88] Zhang Y, Mignone J, MacLellan WR (2015). Cardiac regeneration and stem cells. Physiological Reviews.

[ref-89] Zhang T, Wu Y, Hu Z, Xing W, Kun LV, Wang D, Hu N (2021). Small-molecule integrated stress response inhibitor reduces susceptibility to postinfarct atrial fibrillation in rats *via* the inhibition of integrated stress responses. Journal of Pharmacology and Experimental Therapeutics.

[ref-90] Zhang T, Zhao LL, Cao X, Qi LC, Wei GQ, Liu JY, Yan SJ, Liu JG, Li XQ (2014). Bioinformatics analysis of time series gene expression in left ventricle (LV) with acute myocardial infarction (AMI). Gene.

[ref-91] Zhu X, Yang L, Han X, Huang C, Huang G, Wei T, Shu L, Xu J (2023). Oxidized phospholipids facilitate calcific aortic valve disease by elevating ATF4 through the PERK/eIF2α axis. Aging.

[ref-92] Ziaeian B, Fonarow GC (2016). Epidemiology and aetiology of heart failure. Nature Reviews Cardiology.

